# The Relationship between Metabolically Obese Non-Obese Weight and Stroke: The Korea National Health and Nutrition Examination Survey

**DOI:** 10.1371/journal.pone.0160846

**Published:** 2016-08-05

**Authors:** Young-Gyun Seo, Ho-Chun Choi, Belong Cho

**Affiliations:** 1 Department of Family Medicine, Hallym University Sacred Heart Hospital, Anyang, Gyeonggi-do, Korea; 2 Department of Family Medicine, Healthcare System Gangnam Center, Seoul National University Hospital, Seoul, Korea; 3 Department of Family Medicine, Center for Health Promotion and Optimal Aging, Health Promotion Center for Cancer survivor, Seoul National University Hospital, Seoul, Korea; 4 Advanced Institutes of Convergence Technology, Seoul National University, Suwon, Gyeonggi-do, Korea; 5 Institute on Aging, Seoul National University College of Medicine, Seoul, Korea; National Taiwan University College of Medicine, TAIWAN

## Abstract

**Objective:**

Both metabolic syndrome (MetS) and obesity increase the risk of stroke. However, few studies have compared the risks of stroke associated with metabolically obese non-obese weight (MONW) and metabolically healthy obesity (MHO). This study aimed to compare the prevalence of stroke in MONW and MHO individuals.

**Methods:**

A total of 25,744 subjects aged ≥40 years were selected from the 2007–2014 Korean National Health and Nutrition Examination Survey. MetS was defined using 2001 National Cholesterol Education Program/Adult Treatment Panel III and 2005 American Heart Association/National Heart, Lung, and Blood Institute criteria. Non-obese weight and obesity were defined as a body mass index (BMI) <25 kg/m^2^ and ≥25 kg/m^2^, respectively. MONW was defined as meeting the MetS criteria with a BMI <25 kg/m^2^ and MHO was defined as not meeting the MetS criteria with a BMI ≥25 kg/m^2^.

**Results:**

Women with MONW had a higher prevalence of stroke than those with MHO (odds ratio [OR] = 2.27, 95% confidence interval [CI]: 1.45–3.57). The prevalence of stroke increased as the number of MetS components increased. The ORs for MONW with 3, 4, and 5 MetS components were 1.95 (95% CI: 1.19–3.21), 2.49 (95% CI: 1.46–4.24) and 2.74 (95% CI: 1.39–5.40), respectively.

**Conclusions:**

Our study findings may better emphasize the risk of stroke among more lean but unhealthy individuals, who appear healthy but may be suffering from MetS. These findings also highlight the need for stroke risk factor assessment in non-obese weight individuals.

## Introduction

Both obesity and metabolic syndrome (MetS) are well known to increase the risks of coronary artery disease (CAD), stroke, and mortality.

Regarding the associations of obesity with CAD, stroke, and mortality, a prospective cohort study found that overweight and obese individuals without MetS had an increased risk of cardiovascular events and death compared to normal weight individuals without MetS (hazard ratio [HR]: 1.52, 95% confidence interval [CI]: 1.28–1.80; HR: 1.95, 95% CI: 1.14–3.34, respectively) [1], and another prospective cohort study reported that obesity might increase the risk of incident CAD even in the presence of a healthy metabolic profile (HR: 3.08, 95% CI: 1.10–8.68) [2]. Meanwhile, a recent study demonstrated that obesity is also a risk factor for young-onset ischemic stroke (odds ratio [OR]: 1.57, 95% CI: 1.28–1.94) [3], and another study reported that obesity, even in the absence of overt metabolic aberrations, is associated with an increased all-cause mortality risk (HR: 2.80, 95% CI: 1.18–6.65) [4].

Several studies have addressed the associations of MetS with CAD, stroke, and mortality. For example, two studies found that MetS is associated with the prevalence and severity of CAD (OR: 1.44, 95% CI: 1.09–1.91) [5], as well as stroke (OR: 2.27, 95% CI: 1.80–2.87) [6]. A prospective cohort study further demonstrated that MetS is associated with increased risks of both ischemic and hemorrhagic stroke (HR: 1.65, 95% CI: 1.43–2.82; HR: 1.42, 95% CI: 1.31–2.71, respectively), and that survival rates decreased progressively as the number of MetS components increased [7]. In addition, another study identified MetS as an independent risk factor for acute ischemic non-cardioembolic stroke (OR: 2.39, 95% CI: 1.14–4.98) and noted an increase in the stroke risk as the number of MetS components increased [8]. Two prospective cohort studies found associations of MetS with an increased risk for ischemic stroke (OR: 5.15, 95% CI: 1.86–14.28) [9] and an increase in expanded cardiovascular disease (CVD)-related (CVD plus diabetes) mortality (HR: 1.27, 95% CI: 1.10–1.46) [10].

While several studies have analyzed individual effects of obesity and MetS on CAD, stroke, and mortality, studies comparing metabolically obese non-obese weight (MONW) with metabolically healthy obesity (MHO) are rare. Of these, a recent prospective cohort study found that MONW was associated with increased arterial stiffness and carotid atherosclerosis relative to MHO (OR: 2.98, 95% CI: 1.54–5.73) [11]. Another prospective cohort study found that elderly individuals with MONW exhibited greater all-cause mortality, compared to those with MHO (HR: 1.8, 95% CI: 1.2–2.7) [12]. To address the overall lack of comparison of stroke risks between subjects with MONW and MHO, this study used data from the Korea National Health and Nutrition Examination Survey (KNHANES) and compared the stroke prevalence associated with MONW and MHO to assess whether stroke prevalence among non-obese weight subjects with MetS was higher than those with simple obesity, and further, to determine the need for stroke risk factor assessment in non-obese weight individuals.

## Materials and Methods

### Study subjects

Data were obtained from the fourth, fifth, and sixth KNHANES, a cross-sectional and nationally representative survey conducted between 2007 and 2014 by the Korea Centers for Disease Control and Prevention (KCDC). From among 65,973 individuals, 25,744 were ≥40 years of age and 11,342 were <40 years of age after excluding individuals with incomplete survey records or missing examination results. Among individuals who were <40 years of age, those who reported having had a stroke were only 10 (0.09%). Because of this low stroke incidence, individuals aged less than 40 years were excluded and 25,744 individuals ≥40 years of age were enrolled. All procedures performed were in accordance with the ethical standards of the KCDC (Institutional Review Board numbers: 2007-02CON-04-P, 2008-04EXP-01-C, 2009-01CON-03-2C, 2010-02CON-21-C, 2011-02CON-06-C, 2012-01EXP-01-2C, 2013-07CON-03-4C and 2013-12EXP-03-5C), and the 1964 Helsinki declaration and its later amendments or comparable ethical standards. Signed Informed consent was obtained from all individual participants included in this study. The study protocol does not require any further institutional review board approval because the KNHANES data are publicly available.

### Definition of variables

2001 National Cholesterol Education Program (NCEP) / Adult Treatment Panel III (ATP III) [13] and 2005 American Heart Association (AHA) / National Heart, Lung, and Blood Institute (NHLBI) [14] definition of MetS was used. MetS components are: 1) waist circumference (WC) ≥90 cm in men and ≥80 cm in women (Asian-specific waist circumference cut-offs were based on 2006 International Diabetes Federation definition [15]); 2) serum triglyceride (TG) level ≥150 mg/dL or drug treatment for elevated TG; 3) serum high-density lipoprotein (HDL) cholesterol level <40 mg/dL in men and <50 mg/dL in women, or drug treatment for low HDL cholesterol; 4) blood pressure (BP) ≥130/85 mm Hg, or drug treatment for elevated BP; 5) fasting plasma glucose level ≥100 mg/dL, or drug treatment for elevated blood glucose. The World Health Organization proposed body mass index (BMI) cut-off points of 25 kg/m^2^ for obesity in adult Asians [16]. So we defined non-obese weight and obesity as a BMI <25 and ≥25 kg/m^2^, respectively. MONW was defined as meeting the MetS criteria with a BMI <25 kg/m^2^; MHO was defined as not meeting the MetS criteria with a BMI ≥25 kg/m^2^. The same method was used to define metabolically healthy non-obese weight (MHNW) and metabolically obese obesity (MOO). 2006 International Diabetes Federation (IDF) definition of MetS called MetS-IDF was also used. MONW-IDF, MHO-IDF, MHNW-IDF, and MOO-IDF were defined same manner by using MetS-IDF criteria.

For a sex-based analysis of general subject characteristics, data on the following variables were collected: age, BMI, WC, average monthly household income, education level (≤elementary school, middle or high school, and ≥college), daily total energy intake, daily proportionate energy intake from carbohydrate, protein, and fat, smoking status (never, past, and current), and daily alcohol consumption (one standard drink = 14 g of pure alcohol, adopted from the National Institute on Alcohol Abuse and Alcoholism). The nutritional survey of KNHANES was conducted by trained dietitians using the 24-h dietary recall method. Daily total energy and macro-nutrients intake were calculated using a food composition table published by the Rural Development Administration of Korea [17]. The contribution of each macro-nutrient (carbohydrate, protein, and fat) to energy intake was calculated as the ratio of calories from each macro-nutrient to daily total energy intake. Physical activity was defined according to the metabolic equivalent (MET) and physical activity level (low, moderate, and high), based on the International Physical Activity Questionnaire data processing and analysis guidelines [18]. A history of stroke was based on a self-reported questionnaire asking if the participants had ever been diagnosed with stroke. So the stroke variable was defined as a self-reported physician's diagnosis of stroke.

### Statistical analysis

STATA ver. 13.1 (StataCorp., College Station, TX, USA) was used for data analysis; statistical significance was defined as a P-value <0.05. Student’s t-test and Pearson’s chi-squared test were used for the comparative sex-based analysis of general subject characteristics. A logistic regression model was used to compare the stroke prevalence according to obesity and MetS, as well as to compare the prevalence of stroke in MONW and MHO individuals. Also, the stroke prevalence in sub-groups that had any 3, 4 or all 5 of the MetS components was assessed. Further, a logistic regression model was used to compare the stroke prevalence according to abdominal obesity alone, as well as to compare the prevalence of stroke in MONW-IDF and MHO-IDF individuals. Adjusted ORs were calculated after adjusting for age and sex or for age, sex, smoking status, daily alcohol consumption, physical activity level, and daily total energy intake. Subjects aged 40–65 years (267 reported having had a stroke among 17,190; 1.6%) differed from elderly subjects aged ≥65 years (504 reported having had a stroke among 8,554; 5.9%) on the stroke prevalence (p<0.001). We further calculated adjusted ORs in elderly subjects (age ≥65) who had high prevalence rate of stroke.

## Results

### Subject characteristics

The response rate to the KNHANES IV is 78.4%, the KNHANES V is 80.8%, the KNHANES VI (2013) is 79.3%, and the KNHANES VI (2014) is 77.8%. Characteristics of respondents with and without stroke are presented by sex in S1 and S2 Tables. The 25,744 subjects included 10,646 men (393 reported having had a stroke, 3.7%) and 15,098 women (378 reported having had a stroke, 2.5%). Those who reported having had a stroke differed from those who did not on all of the variables considered, except BMI and obesity in men and proportion of energy from protein in women.

### Stroke prevalence according to obesity and MetS

Stroke prevalence according to obesity and MetS are shown in Table 1. The prevalence of stroke was higher among obese individuals versus non-obese weight individuals, even after adjusting for the potential confounding factors of age, sex, smoking status, daily alcohol consumption, physical activity level, and daily total energy intake (OR: 1.40, 95% CI: 1.21–1.63). After disaggregating data by sex, the respective ORs for men and women after adjusting for potential confounding factors were 1.40 (95% CI: 1.13–1.74) and 1.39 (95% CI: 1.13–1.72). Individuals with MetS had an increased stroke prevalence, compared to those without MetS, even after adjusting for potential confounding factors (OR: 2.08, 95% CI: 1.79–2.42). In a sex-disaggregated analysis, the stroke prevalence remained higher in patients with MetS after adjusting for potential confounding factors (OR: 1.88, 95% CI: 1.53–2.30 for men; OR: 2.39, 95% CI: 1.90–3.01 for women).

**Table 1:** Stroke prevalence according to obesity and metabolic syndrome. OR, odds ratio; CI, confidence interval; MetS, metabolic syndrome.

	Unadjusted		Adjusted^a^		Adjusted^b^	
	OR	(95% CI)	OR	(95% CI)	OR	(95% CI)
Obese vs. Non-obese weight						
Total	1.28	(1.10–1.48)	1.40	(1.20–1.62)	1.40	(1.21–1.63)
Male	1.05	(0.85–1.30)	1.38	(1.11–1.72)	1.40	(1.13–1.74)
Female	1.55	(1.26–1.90)	1.42	(1.15–1.74)	1.39	(1.13–1.72)
MetS vs. No MetS						
Total	2.51	(2.17–2.91)	2.06	(1.77–2.39)	2.08	(1.79–2.42)
Male	1.90	(1.55–2.32)	1.84	(1.50–2.26)	1.88	(1.53–2.30)
Female	3.66	(2.93–4.57)	2.42	(1.92–3.05)	2.39	(1.90–3.01)

^a^ Adjusted for age and sex overall; Adjusted for age in male and female subgroups.

^b^ Adjusted for age, sex, smoking status, daily alcohol consumption, physical activity level, and daily total energy intake overall; Adjusted for all but sex in male and female subgroups.

### Stroke prevalence according to MONW and MHO

Table 2 compares the stroke prevalence of MONW (4,139 individuals [16.1%]; 1,516 men [14.2%]; 2,623 women, [17.4%]), who satisfied 2001 NCEP / ATP III and 2005 AHA / NHLBI definition of MetS, and MHO (3,383 individuals [13.1%]; 1,516 men [14.2%]; 1,867 women [12.4%]). After adjusting for potential confounding factors, the overall stroke prevalence was higher with MONW, compared to MHO (OR: 1.71, 95% CI: 1.28–2.28). This remained significant for women (OR: 2.27, 95% CI: 1.45–3.57), whereas for men, the differences were not statistically significant after adjusting for potential confounding factors. In the ≥65 years age group, the stroke prevalence was higher with MONW than MHO only among women, even after adjusting for potential confounding factors (OR: 2.11, 95% CI: 1.13–3.94).

**Table 2:** Stroke prevalence according to MONW and MHO. OR, odds ratio; CI, confidence interval; MONW, metabolically obese non-obese weight; MHO metabolically healthy obesity.

	Unadjusted		Adjusted^a^		Adjusted^b^	
	OR	(95% CI)	OR	(95% CI)	OR	(95% CI)
MONW vs. MHO (age ≥40 years)						
Total	2.62	(2.00–3.44)	1.68	(1.26–2.24)	1.71	(1.28–2.28)
Male	2.28	(1.59–3.27)	1.34	(0.92–1.96)	1.40	(0.95–2.06)
Female	3.42	(2.23–5.25)	2.27	(1.45–3.55)	2.27	(1.45–3.57)
MONW vs. MHO (age ≥65 years)						
Total	1.39	(0.98–1.99)	1.42	(0.99–2.04)	1.43	(0.99–2.06)
Male	1.18	(0.75–1.85)	1.12	(0.71–1.76)	1.14	(0.72–1.82)
Female	2.18	(1.18–4.04)	2.08	(1.12–3.87)	2.11	(1.13–3.94)

^a^ Adjusted for age and sex overall; Adjusted for age in male and female subgroups.

^b^ Adjusted for age, sex, smoking status, daily alcohol consumption, physical activity level, and daily total energy intake overall; Adjusted for all but sex in male and female subgroups.

### Stroke prevalence according to abdominal obesity

Stroke prevalence according to abdominal obesity alone is shown in Table 3. The prevalence of stroke was higher among abdominal obese individuals versus non abdominal obese individuals, even after adjusting for the potential confounding factors of age, sex, smoking status, daily alcohol consumption, physical activity level, and daily total energy intake (OR: 1.46, 95% CI: 1.25–1.70). After disaggregating data by sex, the respective ORs for men and women after adjusting for potential confounding factors were 1.31 (95% CI: 1.06–1.63) and 1.61 (95% CI: 1.29–2.02). Individuals with MetS-IDF had an increased stroke prevalence, compared to those without MetS-IDF, even after adjusting for potential confounding factors (OR: 1.80, 95% CI: 1.55–2.10). In a sex-disaggregated analysis, the stroke prevalence remained higher in patients with MetS-IDF after adjusting for potential confounding factors (OR: 1.54, 95% CI: 1.22–1.93 for men; OR: 2.08, 95% CI: 1.68–2.58 for women).

**Table 3:** Stroke prevalence according to abdominal obesity. OR, odds ratio; CI, confidence interval; AO, abdominal obesity; MetS-IDF, metabolic syndrome (2006 International Diabetes Federation definition).

	Unadjusted		Adjusted^a^		Adjusted^b^	
	OR	(95% CI)	OR	(95% CI)	OR	(95% CI)
AO vs. No AO						
Total	1.43	(1.24–1.65)	1.44	(1.23–1.67)	1.46	(1.25–1.70)
Male	1.29	(1.04–1.59)	1.29	(1.04–1.60)	1.31	(1.06–1.63)
Female	2.11	(1.69–2.63)	1.62	(1.30–2.03)	1.61	(1.29–2.02)
MetS-IDF vs. No MetS-IDF						
Total	1.99	(1.72–2.30)	1.78	(1.53–2.07)	1.80	(1.55–2.10)
Male	1.50	(1.20–1.88)	1.50	(1.19–1.88)	1.54	(1.22–1.93)
Female	3.02	(2.45–3.72)	2.11	(1.70–2.61)	2.08	(1.68–2.58)

^a^ Adjusted for age and sex overall; Adjusted for age in male and female subgroups.

^b^ Adjusted for age, sex, smoking status, daily alcohol consumption, physical activity level, and daily total energy intake overall; Adjusted for all but sex in male and female subgroups.

### Stroke prevalence according to MONW-IDF and MHO-IDF

Table 4 compares the stroke prevalence of MONW-IDF (2,276 individuals [8.8%]; 442 men [4.2%]; 1,834 women, [12.2%]), who satisfied 2006 IDF definition of MetS, and MHO-IDF (3,832 individuals [14.9%]; 1,900 men [17.9%]; 1,932 women [12.8%]). After adjusting for potential confounding factors, the overall stroke prevalence was higher with MONW-IDF, compared to MHO-IDF (OR: 1.57, 95% CI: 1.13–2.17). This remained significant for women (OR: 2.09, 95% CI: 1.33–3.28), whereas for men, the differences were not statistically significant after adjusting for potential confounding factors. In the ≥65 years age group, the stroke prevalence was higher with MONW-IDF than MHO-IDF only among women, even after adjusting for potential confounding factors (OR: 1.97, 95% CI: 1.08–3.59).

**Table 4:** Stroke prevalence according to MONW-IDF and MHO-IDF. OR, odds ratio; CI, confidence interval; MONW-IDF, metabolically obese non-obese weight (2006 International Diabetes Federation definition); MHO-IDF, metabolically healthy obesity (2006 International Diabetes Federation definition).

	Unadjusted		Adjusted^a^		Adjusted^b^	
	OR	(95% CI)	OR	(95% CI)	OR	(95% CI)
MONW-IDF vs. MHO-IDF (age ≥40 years)						
Total	2.29	(1.73–3.04)	1.54	(1.12–2.12)	1.57	(1.13–2.17)
Male	2.15	(1.35–3.41)	1.07	(0.65–1.76)	1.12	(0.68–1.87)
Female	3.27	(2.14–5.00)	2.06	(1.32–3.23)	2.09	(1.33–3.28)
MONW-IDF vs. MHO-IDF (age ≥65 years)						
Total	1.24	(0.86–1.79)	1.41	(0.95–2.09)	1.47	(0.98–2.19)
Male	1.14	(0.65–2.00)	1.02	(0.58–1.82)	1.14	(0.62–2.07)
Female	2.05	(1.14–3.71)	1.94	(1.07–3.51)	1.97	(1.08–3.59)

^a^ Adjusted for age and sex overall; Adjusted for age in male and female subgroups.

^b^ Adjusted for age, sex, smoking status, daily alcohol consumption, physical activity level, and daily total energy intake overall; Adjusted for all but sex in male and female subgroups.

### Stroke prevalence according to sub-groups of MONW and MHO

Table 5 demonstrates individual comparisons of stroke prevalence in 3 sub-groups of MONW that satisfied 3 of 5 MetS components (2,624 overall [10.2%]; 1,074 men [10.0%]; 1,550 women [10.3%]), 4 of 5 components (1,177 overall [4.6%]; 392 men [3.7%], 785 women [5.2%]), and all 5 components (338 overall [1.3%]; 50 men [0.5%]; 288 women [1.9%]) with the stroke prevalence associated with MHO. Compared to MHO, all 3 MONW overall sub-groups had a higher stroke prevalence after adjusting for potential confounding factors (OR: 1.56, 95% CI: 1.14–2.14 with 3 components; OR: 1.76, 95% CI: 1.22–2.54 with 4 components; and OR: 1.98, 95% CI: 1.13–3.45 with all 5 components). This remained significant for women (OR: 1.95, 95% CI: 1.19–3.21 with 3 components; OR: 2.49, 95% CI: 1.46–4.24 with 4 components; and OR: 2.74, 95% CI: 1.39–5.40 with all 5 components), whereas for men, the differences were not statistically significant. In the ≥65 years age group, only women who satisfied ≥4 components had a higher prevalence of stroke relative to MHO after adjusting for potential confounding factors (OR: 2.70, 95% CI: 1.36–5.38 with 4 components and OR: 2.78, 95% CI: 1.19–6.48 with all 5 components).

**Table 5:** Stroke prevalence according to sub-groups of MONW and MHO. OR, odds ratio; CI, confidence interval; MONW, metabolically obese non-obese weight; MHO metabolically healthy obesity.

	Unadjusted		Adjusted^a^		Adjusted^b^	
	OR	(95% CI)	OR	(95% CI)	OR	(95% CI)
MONW vs. MHO (age ≥40 years)						
Sub-group I ^c^						
Total	2.33	(1.74–3.14)	1.51	(1.11–2.06)	1.56	(1.14–2.14)
Male	2.17	(1.48–3.20)	1.31	(0.88–1.96)	1.37	(0.91–2.07)
Female	2.75	(1.72–4.39)	1.89	(1.16–3.10)	1.95	(1.19–3.21)
Sub-group II ^d^						
Total	3.04	(2.17–4.25)	1.80	(1.25–2.58)	1.76	(1.22–2.54)
Male	2.51	(1.55–4.09)	1.30	(0.78–2.19)	1.29	(0.76–2.21)
Female	4.20	(2.57–6.88)	2.55	(1.50–4.33)	2.49	(1.46–4.24)
Sub-group III ^e^						
Total	3.41	(2.10–5.53)	1.88	(1.09–3.24)	1.98	(1.13–3.45)
Male	2.84	(0.98–8.24)	1.17	(0.38–3.55)	1.34	(0.43–4.24)
Female	5.00	(2.73–9.16)	2.56	(1.31–5.00)	2.74	(1.39–5.40)
MONW vs. MHO (age ≥65 years)						
Sub-group I ^c^						
Total	1.31	(0.89–1.92)	1.32	(0.89–1.95)	1.35	(0.91–2.01)
Male	1.19	(0.74–1.93)	1.15	(0.71–1.87)	1.23	(0.75–2.01)
Female	1.76	(0.91–3.42)	1.70	(0.87–3.31)	1.77	(0.89–3.52)
Sub-group II ^d^						
Total	1.55	(1.01–2.36)	1.58	(1.02–2.43)	1.52	(0.98–2.36)
Male	1.13	(0.62–2.06)	1.03	(0.56–1.90)	0.94	(0.50–1.78)
Female	2.73	(1.39–5.36)	2.60	(1.32–5.14)	2.70	(1.36–5.38)
Sub-group III ^e^						
Total	1.42	(0.79–2.56)	1.79	(0.93–3.42)	1.88	(0.95–3.72)
Male	1.23	(0.35–4.31)	1.01	(0.28–3.65)	1.17	(0.29–4.70)
Female	2.60	(1.18–5.73)	2.37	(1.06–5.31)	2.78	(1.19–6.48)

^a^ Adjusted for age and sex overall; Adjusted for age in male and female subgroups.

^b^ Adjusted for age, sex, smoking status, daily alcohol consumption, physical activity level, and daily total energy intake overall; Adjusted for all but sex in male and female subgroups.

^c^ MONW that satisfied 3 of 5 metabolic components.

^d^ MONW that satisfied 4 of 5 metabolic components.

^e^ MONW that satisfied all 5 metabolic components.

## Discussion

In this study, we assessed the need for stroke risk factor assessment in non-obese weight individuals by comparing not only the stroke prevalence based on the presence or absence of obesity and MetS, which were suggested as risk factors in previous studies, but also by comparing MONW and MHO, thus controlling for obesity in MetS and for MetS in obesity, to individually examine the pure effects of MetS and obesity.

Obese subjects had a statistically significantly higher stroke prevalence than non-obese weight subjects, both overall and in sex-based subgroup analyses. This was consistent with previous study results [3] and suggests that stroke prevention and screening are necessary for obese individuals.

Similarly, subjects with MetS had a statistically significantly higher stroke prevalence than those without MetS, irrespective of sex. This was also consistent with previous study results [6–9], and suggests the need for stroke prevention and screening in individuals with MetS.

As mentioned previously, we also compared stroke prevalence in MONW and MHO subjects. The stroke prevalence among not only subjects with MONW who satisfied 2001 NCEP / ATP III and 2005 AHA / NHLBI definition of MetS but also those who satisfied 2006 IDF definition of MetS was statistically significantly higher than that among overall and female subjects with MHO. In women, the stroke prevalence with MONW was statistically significantly higher than with MHO, even at an age ≥65 years. Two prospective cohort studies from Japan found an elevated stroke risk in women with MetS [19, 20], whereas other studies concluded that the effects of MetS on CVD might be greater in women than in men [21–23]. Although sex-based differences in MetS diagnostic criteria might have contributed to the observed differences, this study followed the Asian WC criteria, and the prevalence of MetS tended to be higher in women (34.8% in men and 39.8% in women, *p* <0.001), contrary to previous studies that used the Japanese MetS diagnostic criteria [19, 20]. Therefore, it is difficult to assume that the inclusion of only extremely obese women with MetS would greatly influence the increase in stroke prevalence. Moreover, MetS might be attributable to sex-based differences in cholesterol levels, as previous studies have found that women with MetS have a higher total cholesterol level than women without MetS. This study showed statistically significant differences in total cholesterol only in women with MetS, compared to those without MetS (188.3 mg/dL versus 187.5 mg/dL in men, *p* = 0.322; 198.6 mg/dL versus 195.4 mg/dL in women, *p* <0.001). Therefore, differences in cholesterol levels may explain the observed sex-based differences in stroke prevalence.

In addition, studies have demonstrated that the effects of diabetes or inflammatory activity on stroke, measured using high-sensitivity C-reactive protein levels, and circulating adiponectin levels might differ between men and women [22–24], and the combined effects of all aforementioned factors might be attributable to the sex-based differences shown in this study.

Previous Japanese studies were based on Japanese MetS diagnostic criteria in which central obesity was a mandatory criterion and indicated that MetS increases the stroke risk only in women [19, 20]. Meanwhile, the risks of CVD were shown to increase with an increasing WC [25, 26], whereas the previous Japanese studies considered a standard WC of 90 cm in women, the present study employed a stricter standard WC of 80 cm for central obesity. We also analyzed the stroke prevalence using IDF definition of MetS which central obesity was a mandatory criterion, and same results were derived. Hence, the higher stroke prevalence demonstrated in this study in response to a stricter standard is significant. Accordingly, individual MetS factors in addition to central obesity might play a role in the increased stroke prevalence, and we therefore investigated whether changes in the number of satisfied MetS diagnostic criteria would also influenced the stroke prevalence.

The MHO-associated stroke prevalence was compared with the prevalence of 3 MONW sub-groups (subjects with MONW that satisfied 3, 4, and all 5 MetS diagnostic criteria used in this study). Overall, the stroke prevalence of all 3 MONW sub-groups was statistically significantly higher than that of the MHO group. This remained significant for women. In other words, the stroke prevalence increased when the number of MetS components increased (P for trend <0.001 in the ≥40 years age group; 0.004 in the ≥65 years age group), suggesting that the dose-response relationship demonstrated earlier for MetS is also applicable to MONW [8].

This study was based on KNHANES questionnaires, and thus the results might have been affected by recall bias. Further, this cross-sectional study was limited in its ability to demonstrate causal relationships for stroke risk. Most of missing data came from pediatric data such as past medical history, smoking, alcohol related data, and laboratory test results. Still potential sample bias may exist because of other missing data, but the results of this study can be generalized to the Korean population as a whole because of a high response rate (about 80%), the large population sample size, and a proportional systematic sampling with multistage stratification based on geographical area, sex, and age group. This study was significant because it used a large-scale, nationally representative dataset collected over a period of 8 years. Moreover, another strength of this study was the inclusion of analyses adjusted for lifestyle factors, such as smoking, alcohol, physical activity, and nutrition.

Our study demonstrated the prevalence associated individually with MetS and obesity, as well as the relative degree of associations by comparing prevalence while controlling for obesity in MetS and for MetS in obesity, unlike previous studies that only investigated whether MetS or obesity individually increased the stroke risk. Additionally, we demonstrated that MONW was more closely associated with an increased stroke prevalence than MHO, and that a positive dose-response relationship between MetS components and stroke prevalence also existed for MONW. Future prospective studies that evaluate and compare the stroke risks associated with MONW and MHO are needed to prove this causality and dose-response relationship.

## Conclusions

MetS, which is not externally visible, may be more dangerous than the more visible obesity phenotype. Therefore, our study findings may better emphasize the risk of stroke among more lean but unhealthy individuals, who appear healthy but may be suffering from MetS. These findings also highlight the need for stroke risk factor assessment in non-obese weight individuals. The stroke risk factor assessment should be undertaken in non-obese weight patients who already had cardiovascular risk factors such as old age or family history of premature coronary heart disease. It would be helpful to consider measuring waist circumference, checking BP, and asking about current medications as an initial screening tool. This study is anticipated to serve as a basis for future prospective studies on stroke risk assessment and comparison in MONW and MHO.

## Supporting Information

S1 Table Male subject characteristics (n = 10,646). (DOCX)

S2 Table Female subject characteristics (n = 15,098). (DOCX)
