# Supplementary material for: The Relationship between Metabolically Obese Non-Obese Weight and Stroke: The Korea National Health and Nutrition Examination Survey
Source: PLoS One. 2016 Aug 5;11(8):e0160846. doi: 10.1371/journal.pone.0160846 (PMC4975497; doi:10.1371/journal.pone.0160846)
Supplement: S1 Table — (DOCX) [file pone.0160846.s001.docx]

**S1 Table. Male subject characteristics (n = 10,646)**

| Characteristic | | Total (n = 10,646) Mean±SD or number (%) | Stroke (n = 393) Mean±SD or number (%) | No stroke (n = 10,253) Mean±SD or number (%) | P-value^a^ |
| --- | --- | --- | --- | --- | --- |
| Age (years) |  | 58.9±11.5 | 67.1±9.2 | 58.6±11.5 | <0.001 |
| BMI (kg/m^2^) |  | 23.9±3.0 | 24.1±3.0 | 23.9±3.0 | 0.343 |
| Waist circumference (cm) |  | 85.2±8.5 | 86.7±8.6 | 85.1±8.5 | <0.001 |
| Income (x10^4^ won) |  | 351.6±820.0 | 227.8±417.2 | 356.4±831.2 | 0.002 |
| Education |  |  |  |  | <0.001 |
|  | ≤ Elementary school | 2,479 (23.3) | 151 (38.4) | 2,328 (22.7) |  |
|  | Middle or high school | 5,175 (48.6) | 182 (46.3) | 4,993 (48.7) |  |
|  | ≥ College | 2,992 (28.1) | 60 (15.3) | 2,932 (28.6) |  |
| Nutrient intake |  |  |  |  |  |
|  | Total energy (kcal/day) | 2,218.0±858.5 | 1,872.3±731.4 | 2,231.2±860.2 | <0.001 |
|  | Carbohydrates (% of energy) | 66.1±14.0 | 70.9±12.3 | 65.9±14.0 | <0.001 |
|  | Protein (% of energy) | 13.9±4.1 | 13.2±3.7 | 13.9±4.1 | 0.002 |
|  | Fat (% of energy) | 15.1±7.8 | 13.5±7.7 | 15.2±7.8 | <0.001 |
| Smoking |  |  |  |  | <0.001 |
|  | Never | 1,733 (16.3) | 59 (15.0) | 1,674 (16.3) |  |
|  | Past | 5,109 (48.0) | 230 (58.5) | 4,879 (47.6) |  |
|  | Current | 3,804 (35.7) | 104 (26.5) | 3,700 (36.1) |  |
| Daily alcohol consumption^b^ |  |  |  |  | <0.001 |
|  | None | 3,501 (32.9) | 211 (53.7) | 3,290 (32.1) |  |
|  | Light | 2,859 (26.9) | 92 (23.4) | 2,767 (27.0) |  |
|  | Moderate | 1,667 (15.7) | 42 (10.7) | 1,625 (15.9) |  |
|  | Heavy | 2,619 (24.6) | 48 (12.2) | 2,571 (25.1) |  |
| Level of physical activity^c^ |  |  |  |  | 0.008 |
|  | Low | 3,976 (37.4) | 155 (39.4) | 3,821 (37.3) |  |
|  | Moderate | 3,649 (34.3) | 153 (38.9) | 3,496 (34.1) |  |
|  | High | 3,021 (28.4) | 85 (21.6) | 2,936 (28.6) |  |
| Obesity |  |  |  |  | 0.637 |
|  | Non-obese | 6,945 (65.2) | 252 (64.1) | 6,693 (65.3) |  |
|  | Obese | 3,701 (34.8) | 141 (35.9) | 3,560 (34.7) |  |
| MetS |  |  |  |  | <0.001 |
|  | No | 6,945 (65.2) | 198 (50.4) | 6,747 (65.8) |  |
|  | Yes | 3,701 (34.8) | 195 (49.6) | 3,506 (34.2) |  |
| Metabolic status |  |  |  |  | <0.001 |
|  | MHNW | 5,429 (51.0) | 153 (38.9) | 5,276 (51.5) |  |
|  | MONW | 1,516 (14.2) | 99 (25.2) | 1,417 (13.8) |  |
|  | MHO | 1,516 (14.2) | 45 (11.5) | 1,471 (14.4) |  |
|  | MOO | 2,185 (20.5) | 96 (24.4) | 2,089 (20.4) |  |
| AO |  |  |  |  | 0.020 |
|  | No | 7,599 (71.4) | 260 (66.2) | 7,339 (71.6) |  |
|  | Yes | 3,047 (28.6) | 133 (33.8) | 2,914 (28.4) |  |
| MetS-IDF |  |  |  |  | <0.001 |
|  | No | 8,403 (78.9) | 282 (71.8) | 8,121 (79.2) |  |
|  | Yes | 2,243 (21.1) | 111 (28.2) | 2,132 (20.8) |  |
| Metabolic status |  |  |  |  | 0.001 |
|  | MHNW-IDF | 6,503 (61.1) | 224 (57.0) | 6,279 (61.2) |  |
|  | MONW-IDF | 442 (4.2) | 28 (7.1) | 414 (4.0) |  |
|  | MHO-IDF | 1,900 (17.9) | 58 (14.8) | 1,842 (18.0) |  |
|  | MOO-IDF | 1,801 (16.9) | 83 (21.1) | 1,718 (16.8) |  |

SD, standard deviation; BMI, body mass index (weight in kilograms divided by height in meters squared); MetS, metabolic syndrome; MHNW, metabolically healthy normal weight; MONW, metabolically obese normal weight; MHO, metabolically healthy obesity; MOO, metabolically obese obesity; AO, abdominal obesity; MetS-IDF, metabolic syndrome (2006 International Diabetes Federation definition); MHNW-IDF, metabolically healthy normal weight (2006 International Diabetes Federation definition); MONW-IDF, metabolically obese normal weight (2006 International Diabetes Federation definition); MHO-IDF, metabolically healthy obesity (2006 International Diabetes Federation definition); MOO-IDF, metabolically obese obesity (2006 International Diabetes Federation definition).

^a^ *P* value from a T test for continuous outcomes or χ2 test for binary outcomes, comparing differences between any 2 study groups.

^b^ None, <1; Light, 1–14.99; Moderate, 15–29.99; Heavy, ≥30 g/day.

^c^ Categorical variable from the International Physical Activity Questionnaire Research Committee.
