# Supplementary material for: The Relationship between Metabolically Obese Non-Obese Weight and Stroke: The Korea National Health and Nutrition Examination Survey
Source: PLoS One. 2016 Aug 5;11(8):e0160846. doi: 10.1371/journal.pone.0160846 (PMC4975497; doi:10.1371/journal.pone.0160846)
Supplement: S2 Table — (DOCX) [file pone.0160846.s002.docx]

**S2 Table. Female subject characteristics (n = 15,098)**

| Characteristic | | Total (n = 15,098) Mean±SD or number (%) | Stroke (n = 378) Mean±SD or number (%) | No stroke (n = 14,720) Mean±SD or number (%) | P-value^a^ |
| --- | --- | --- | --- | --- | --- |
| Age (years) |  | 58.2±11.6 | 67.0±9.3 | 57.9±11.6 | <0.001 |
| BMI (kg/m^2^) |  | 24.0±3.2 | 24.9±3.5 | 24.0±3.2 | <0.001 |
| Waist circumference (cm) |  | 81.0±9.3 | 85.1±9.9 | 80.9±9.3 | <0.001 |
| Income (x10^4^ won) |  | 335.7±946.3 | 161.5±191.7 | 340.1±957.4 | <0.001 |
| Education |  |  |  |  | <0.001 |
|  | ≤ Elementary school | 6,420 (42.5) | 285 (75.4) | 6,135 (41.7) |  |
|  | Middle or high school | 6,416 (42.5) | 81 (21.4) | 6,335 (43.0) |  |
|  | ≥ College | 2,262 (15.0) | 12 (3.2) | 2,250 (15.3) |  |
| Nutrient intake |  |  |  |  |  |
|  | Total energy (kcal/day) | 1,627.6±634.5 | 1,471.9±638.7 | 1,631.6±633.9 | <0.001 |
|  | Carbohydrates (% of energy) | 72.1±11.4 | 75.1±9.7 | 72.1±11.4 | <0.001 |
|  | Protein (% of energy) | 13.6±3.9 | 13.2±3.5 | 13.6±3.9 | 0.052 |
|  | Fat (% of energy) | 14.6±8.1 | 12.0±7.2 | 14.6±8.2 | <0.001 |
| Smoking |  |  |  |  | <0.001 |
|  | Never | 13,936 (92.3) | 336 (88.9) | 13,600 (92.4) |  |
|  | Past | 550 (3.6) | 30 (7.9) | 520 (3.5) |  |
|  | Current | 612 (4.1) | 12 (3.2) | 600 (4.1) |  |
| Daily alcohol consumption^b^ |  |  |  |  | <0.001 |
|  | None | 11,317 (75.0) | 320 (84.7) | 10,997 (74.7) |  |
|  | Light | 2,874 (19.0) | 50 (13.2) | 2,824 (19.2) |  |
|  | Moderate | 602 (4.0) | 2 (0.5) | 600 (4.1) |  |
|  | Heavy | 305 (2.0) | 6 (1.6) | 299 (2.0) |  |
| Level of physical activity^c^ |  |  |  |  | 0.001 |
|  | Low | 6,672 (44.2) | 201 (53.2) | 6,471 (44.0) |  |
|  | Moderate | 5,433 (36.0) | 125 (33.1) | 5,308 (36.1) |  |
|  | High | 2,993 (19.8) | 52 (13.8) | 2,941 (20.0) |  |
| Obesity |  |  |  |  | <0.001 |
|  | Non-obese | 9,842 (65.2) | 208 (55.0) | 9,634 (65.5) |  |
|  | Obese | 5,256 (34.8) | 170 (45.0) | 5,086 (34.6) |  |
| MetS |  |  |  |  | <0.001 |
|  | No | 9,086 (60.2) | 113 (29.9) | 8,973 (61.0) |  |
|  | Yes | 6,012 (39.8)) | 265 (70.1) | 5,747 (39.0) |  |
| Metabolic status |  |  |  |  | <0.001 |
|  | MHNW | 7,219 (47.8) | 87 (23.0) | 7,132 (48.5) |  |
|  | MONW | 2,623 (17.4) | 121 (32.0) | 2,502 (17.0) |  |
|  | MHO | 1,867 (12.4) | 26 (6.9) | 1,841 (12.5) |  |
|  | MOO | 3,389 (22.5) | 144 (38.1) | 3,245 (22.0) |  |
| AO |  |  |  |  | <0.001 |
|  | No | 7,124 (47.2) | 114 (30.2) | 7,101 (47.6) |  |
|  | Yes | 7,974 (52.8) | 264 (69.8) | 7,710 (52.4) |  |
| MetS-IDF |  |  |  |  | <0.001 |
|  | No | 9,940 (65.8) | 150 (39.7) | 9,790 (66.5) |  |
|  | Yes | 5,158 (34.2) | 228 (60.3) | 4,930 (33.5) |  |
| Metabolic status |  |  |  |  | <0.001 |
|  | MHNW-IDF | 8,008 (53.0) | 121 (32.0) | 7,887 (53.6) |  |
|  | MONW-IDF | 1,834 (12.2) | 87 (23.0) | 1,747 (11.9) |  |
|  | MHO-IDF | 1,932 (12.8) | 29 (7.7) | 1,903 (12.9) |  |
|  | MOO-IDF | 3,324 (22.0) | 141 (37.3) | 3,183 (21.6) |  |

SD, standard deviation; BMI, body mass index (weight in kilograms divided by height in meters squared); MetS, metabolic syndrome; MHNW, metabolically healthy normal weight; MONW, metabolically obese normal weight; MHO, metabolically healthy obesity; MOO, metabolically obese obesity; AO, abdominal obesity; MetS-IDF, metabolic syndrome (2006 International Diabetes Federation definition); MHNW-IDF, metabolically healthy normal weight (2006 International Diabetes Federation definition); MONW-IDF, metabolically obese normal weight (2006 International Diabetes Federation definition); MHO-IDF, metabolically healthy obesity (2006 International Diabetes Federation definition); MOO-IDF, metabolically obese obesity (2006 International Diabetes Federation definition).

^a^ *P* value from a T test for continuous outcomes or χ2 test for binary outcomes, comparing differences between any 2 study groups.

^b^ None, <1; Light, 1–14.99; Moderate, 15–29.99; Heavy, ≥30 g/day.

^c^ Categorical variable from the International Physical Activity Questionnaire Research Committee.
